# Perceptions of ‘Precision’ and ‘Personalised’ Medicine in Singapore and Associated Ethical Issues

**DOI:** 10.1007/s41649-021-00165-3

**Published:** 2021-03-20

**Authors:** Serene Ong, Jeffrey Ling, Angela Ballantyne, Tamra Lysaght, Vicki Xafis

**Affiliations:** 1 Centre for Biomedical Ethics, Yong Loo Lin School of Medicine, National University of Singapore, Singapore; 2 Faculty of Science, National University of Singapore, Singapore; 3 Department of Primary Health Care and General Practice, University of Otago, Wellington, New Zealand

**Keywords:** Precision medicine, Data sharing, Social licence, Precision health, Personalised medicine, Big data

## Abstract

Governments are investing in precision medicine (PM) with the aim of improving healthcare through the use of genomic analyses and data analytics to develop tailored treatment approaches for individual patients. The success of PM is contingent upon clear public communications that engender trust and secure the social licence to collect and share large population-wide data sets because specific consent for each data re-use is impractical. Variation in the terminology used by different programmes used to describe PM may hinder clear communication and threaten trust. Language is used to create common understanding and expectations regarding precision medicine between researchers, clinicians and the volunteers. There is a need to better understand public interpretations of PM-related terminology. This paper reports on a qualitative study involving 24 focus group participants in the multi-lingual context of Singapore. The study explored how Singaporeans interpret and understand the terms ‘precision medicine’ and ‘personalised medicine’, and which term they felt more aptly communicates the concept and goals of PM. Results suggest that participants were unable to readily link the terms with this area of medicine and initially displayed preferences for the more familiar term of ‘personalised’. The use of visual aids to convey key concepts resonated with participants, some of whom then indicated preferences for the term ‘precision’ as being a more accurate description of PM research. These aids helped to facilitate dialogue around the ethical and social value, as well as the risks, of PM. Implications for programme developers and policy makers are discussed.

## Introduction

Precision medicine (PM) aims to improve healthcare with the use of genomic analyses and data analytics to develop tailored approaches to predicting disease progression and treatment responses for individual patients (Marquart et al. 2018). This data-intensive approach differs from evidence-based medicine, where the knowledge base depends predominantly on evidence from epidemiological studies and clinical trials that reflect the health status and treatment response of specific populations (Goldberger and Buxton 2013). For the PM paradigm to work, many people must be willing to share clinical data and biological materials with researchers so they can identify patterns and stratify patient characteristics into sub-population groups (Schaefer et al. 2019).

More than 40 countries have invested in PM initiatives (Dana 2017), and PM is one of the nine programme areas identified by the World Economic Forum’s Center for the Fourth Industrial Revolution (National Academies of Sciences, Engineering, and Medicine 2018). For example, the National Institutes of Health (NIH) in the USA has launched *All of US*, which seeks to recruit one million volunteers to share genomic samples and data for research (Genetics Home Reference 2020). The China Precision Medicine Initiative will receive the equivalent of US$9.2 billion in funding over 15 years (Cyranoski 2016; Jia et al. 2015).

In the multi-ethnic context of Singapore, the government has started developing PM with large-scale whole-genome sequencing in the SG10k project and the SingHealth Duke-NUS Institute of PRecISion Medicine (PRISM) (Wu et al. 2019). This research will upscale into the National Precision Medicine initiative from 2021. With an under-representation of Asian genomes in global databases (Bentley et al. 2017; Crider et al. 2006; Lee et al. 2019; Popejoy and Fullerton 2016), this initiative will contribute to greater diversity in genomics and may lead to more tailored treatment approaches appropriate for Asian populations. As of 2016, only 14% of 2511 genome-wide association studies were on Asian ancestry (Popejoy and Fullerton 2016). In contrast, Asian populations account for about 60% of the world’s population (United Nations 2019).

This study explores how Singaporeans understand key terms used to describe PM. Terminology is important because different words carry often subtle connotations and implications that can influence public expectations of science and facilitate or impede a constructive conversation about research. The choice of terms can also influence public acceptance and willingness to participate in research.

Various terms have been put forth to describe the emerging field: precision medicine, personalised medicine, stratified medicine, P4 medicine (Academy of Medical Sciences 2015; Pokorska-Bocci et al. 2014). *Precision* and *personalised medicine* have emerged as favoured terms. While the Human Genome Project initially adopted the term ‘personalised medicine’, there has been a gradual shift towards ‘precision medicine’ (Katsnelson 2013). The shift came from concerns that ‘personalised medicine’ did not adequately convey the novelty of genome data-driven initiatives and might raise unrealistic expectations for personalised medical care and medicines made for individual patients, rather than tailoring therapies for subcategories of disease as defined primarily by genomics (Ashley 2015; Lopez-Campos et al. 2014). Nevertheless, some programmes continue to use ‘personalised medicine’ (e.g. in the European Union) while others use the two terms interchangeably to broadly refer to the same concept.

Both terms describe, in different ways, overlapping visions of future medicine and healthcare driven by data-intensive biomedical research (Erikainen and Chan 2019). Some authors argue that different terminology signals an orientation towards different goals, values and conceptual frameworks (Juengst and McGowan 2018). However, the differences might also reflect perceptions of how research participants understand and respond to the terms. Understanding the social and contextual meaning of these terms, and the ethical connotations they might carry, can help researchers and policy makers develop trust relationships with participants based on transparency and clear communication.

### Trust and Public Communications of Science

Effective communication of the concepts of PM is crucial. In addition to the potential benefits of PM, new risks, obligations for participants and perhaps even new conceptions of ‘the patient’ are emerging. PM moves beyond treating manifested illnesses towards a ‘multilayer characterisation of individuals’ (Eyal et al. 2019). There are already current clinical challenges arising from the introduction of PM to the clinic; for example, the predictive aspect of genetic testing can create ‘patients-in-waiting’ (Timmermans and Buchbinder 2010). Furthermore, PM may diminish the traditional role of the physician as gatekeepers of health information, and new players are being introduced to manage the ‘datafication’ of healthcare (Prainsack 2017). Indeed, by ‘empowering individuals to monitor and take a more active role in their own health’ (Obama 2016), PM is generating new notions of ‘genomic citizenship’ (Sabatello and Appelbaum 2017). However, PM can also create ‘new patterns of exclusion’ in groups who cannot, or will not, participate in the datafication wave (Prainsack 2017).

Effective science communication relies on the provision of accurate and accessible information and public trust. Information about the science is useful in providing the general public and research participants with key understandings so they are able to engage with related issues in greater depth. Nonetheless, researchers should avoid defaulting to the ‘deficit model’, where public communications are framed as a one-way transfer of knowledge from scientists to the public without taking into account the complex role that ethical values play in science communication and policy-making (Bak 2001; Lee et al. 2005; Metcalfe 2019). The deficit model presumes that public skepticism towards science stems from a lack of scientific knowledge. However, this model has been criticised for being overly simplistic (Wright and Nerlich 2006), as it characterises the public as an ignorant, homogenous group that needs educating (Simis et al. 2016). It has also been criticised for focusing on knowledge asymmetries rather than the complex roles that ethics and values play in science policy-making (Bak 2001; Lee et al. 2005). There is also a danger in perceiving the public as a genetic resource to be used, rather than engaging with the public in a partnership (Au 2020).

Prior empirical research suggests that although many people do not fully comprehend the scientific terms and concepts of PM or genomics (Hall et al. 2015; Wynn et al. 2018), there is general public support for these initiatives, including conditional support for data-sharing research programmes based on broad consent (i.e. without a requirement to re-consent each time data is accessed) (Garrison et al. 2016; Milne et al. 2019). In Singapore, similar findings suggest that people with limited understanding of genetics and genomic profiling are willing to participate in data-sharing research under broad consent models when provided with information that is clear, coherent and accessible to them (Bylstra et al. 2017; Lysaght et al. 2020).

PM raises complex ethical and governance challenges and viable responses to these must be tailored to the local ‘cultural preferences and attitudes, available resources, and the wider political landscape’ in which such research is embedded (Minari et al. 2018). One governance challenge is that of genomic sovereignty, or national control over citizens’ genomic information (Benjamin 2009). For instance, China’s *Regulation of Human Genetic Resources* has certain restrictions on the collection and sharing of Chinese genetic material with international entities, both of which require government approval (State Council 2019). Another example is the growing global demand for indigenous data sovereignty and collective (rather than individual) models of consent for medical research (Reardon and TallBear 2012; Walter et al. 2021).

Some studies suggest that different ethnic communities may interpret key terms and concepts differently, which may discourage participation of minority groups (Dang et al. 2014) already under-represented in genome databases (Bentley et al. 2017; Crider et al. 2006; Lee et al. 2019; Popejoy and Fullerton 2016). Differential interpretation will reflect, in part, different histories with medical and genomic research (Sun 2017). Marginalised groups may experience greater distrust in PM research, as these groups have previously been subject to egregious research practices (recall the Tuskegee Syphilis study; (Hodge 2012)), ignored (Nelson 2011) and misrepresented (Vasquez and Deister 2019). Achieving diversity and inclusivity in PM will thus require engaging with different ethnic communities to better understand and integrate their perspectives into communication strategies and governance mechanisms (Kraft et al. 2018; Pratt 2019). Our research supports these efforts with an exploratory study of how Singaporeans interpret and understand the terms ‘precision medicine and ‘personalised medicine’, and which term participants thought more aptly communicates the concept and goals of PM.

## Methods

A full description of the methods has been published in a prior paper which reported results relating to data governance [removed for blinded review]. Here we report results relating to understanding of terminology. The qualitative study design employed focus groups conducted in three of the four major languages spoken in Singapore: English, Malay and Mandarin. Most Singaporeans (73.2%) are literate in two languages: English, and their mother tongue (Mandarin, Malay or Tamil). In 2016, the proportion of the population who spoke only in their mother tongue was 19.4% for Chinese, 9.9% for Malays, and 3.6% for Indians (Singapore Department of Statistics 2016, 17). Based on the paucity of Singaporeans who speak Tamil exclusively, we decided not to conduct a focus group in Tamil. Ethnicity was not an exclusion criterion; ethnic Indian Singaporeans who spoke English were included. It was essential to interview participants in their primary language for two reasons: to effectively test their perceptions of the nuance of terms in different languages, and to facilitate inclusion of participants who are more comfortable communicating in a non-English language.

We contracted a commercial market research firm (Beacon Consulting Pte Limited) with multilingual capabilities to recruit participants from the different ethnic groups and conduct the focus groups in the three languages. The firm also translated the English interview guide into Mandarin and Malay, and checked the translations for accuracy via reverse translation. Translation issues will be discussed in the Results.

Focus groups were conducted at the premises of the market research firm with at least one member of the research team present to observe. The focus groups began with an initial and general discussion of participants’ understandings and interpretations of the terms ‘precision medicine’ and ‘personalised medicine’. Participants were then shown the educational video introducing the basics of PM and what a PM programme would entail.^1^ Two case examples in the video described possible scenarios where PM might be applied/used. Neither of the terms ‘precision’ or ‘personalised’ was used during the video. After viewing the video, participants were asked which term best described the research techniques and strategies shown in the video [removed for blinded review].

The market research firm transcribed the discussions, which lasted between 60 and 90 min, from audio recordings and translated the non-English discussions into English (checked with reverse translation). Transcripts were analysed in NVivo 12 (QSR International) using qualitative thematic analysis (Miles and Huberman 1994). The coding was primarily done by one graduate student (SO), with contributions from VX, and checked by a senior researcher (TL) until thematic saturation was reached (where no new themes relevant to the research aims were emerging). Content was coded inductively for topics, which were progressively collapsed into broader thematic categories, and refined over several meetings between the coders and study team (TL, JL, VX, and AB).

The National University of Singapore (NUS) Institutional Review Board approved the protocol (S-19-045E) for this study and all participants gave their informed consent to participate. A SGD50 supermarket voucher was given to participants who completed the focus groups as a token of appreciation.

## Results

**Fig. 1:**
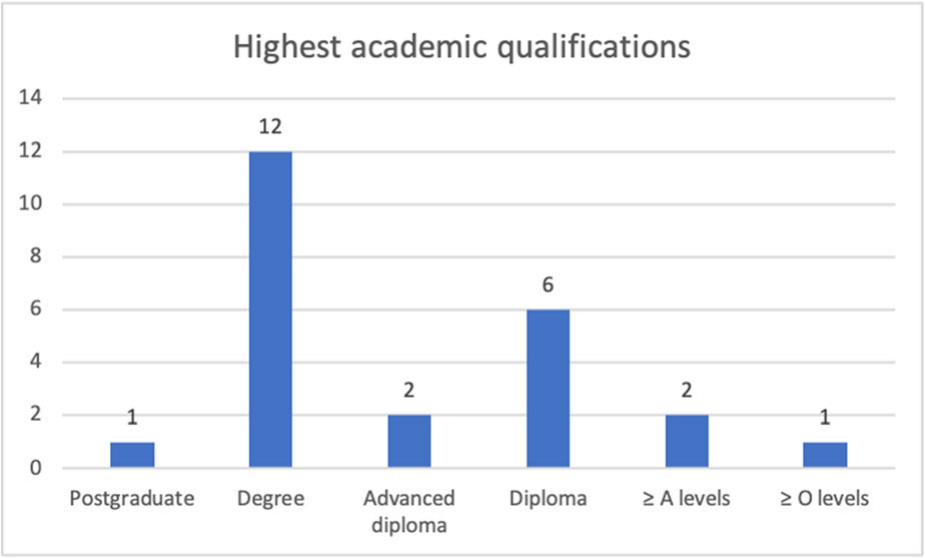
Highest academic qualifications of participants (*n* = 24)

**Fig. 2:**
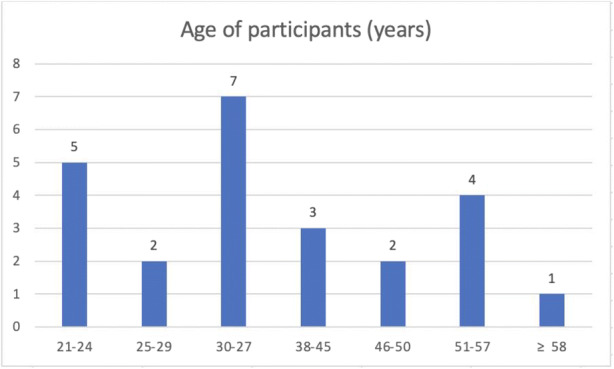
Age of participants (*n* = 24)

From May to July 2019, we conducted three focus group discussions (one each in English, Mandarin and Malay) with 24 participants in total (11 participants in the English group, eight in Mandarin and five in Malay). The participants were relatively well-educated with just over half holding a degree or post graduate qualification (13/24)^2^ (see Fig. 1). The ages of our participants ranged from 21 to ≥ 58 years (Fig. 2).

The following results are organised around (1) participants’ conceptions and understanding of the terms ‘precision’ or ‘personalised medicine’ before and after watching the video, and (2) their views about genomic data sharing.

- Understandings of ‘personalised’ and ‘precision medicine’ before video

Prior to being shown the video, participants discussed their understanding and interpretations of the terms ‘personalised’ and ‘precision’ medicine. Very few participants across the English and Mandarin focus groups said they had heard of ‘precision medicine’ before being invited to the discussions, but more participants were familiar with the term ‘personalised medicine’.

In the Malay-speaking group, none of the participants had heard of either term before. There was some initial confusion with the term precision medicine (‘perubatan kepersisan’) because the term for precision (‘kepersisan’) is not a word that is commonly used. The facilitator explained saying that ‘kepersisan’ is similar to ‘kepepatan’. ‘Kepepatan’ is based on the root word ‘tepat’, which means ‘accurate’. Using the term ‘perubatan ketapatan’ was not appropriate because it implies that current medicine is inaccurate; however, when the facilitator linked the two similar terms ‘kepersisan’ and ‘kepepatan’, participants were better able to understand the meaning of the former.

For participants in the Mandarin and English-speaking focus groups, the term ‘personalised medicine’ was more familiar and understandable than ‘precision medicine’. Personalised medicine sounded ‘more ordinary’ (P7_CFG) and ‘customised to me, as an individual and not the whole population’ (P6_CFG). Personalised medicine was also understood as relating to the standard efforts of a medical team to provide treatments that are appropriate for the patient and in line with general clinical evidence:

My idea is when a person is very sick…the medical team will come up with certain medicines just meant for that person, that particular patient;… there are special types of antibiotics but doesn’t mean that you can accept it, your body, you might reject [the first line drug(s)]…so I think the medical team will … come up with something that is more personalised based on the condition of that particular patient and also maybe the way that she reacted to the drugs (P1_EFG).

On the other hand, precision medicine was interpreted as being more abstract and technical. ‘Sounds very inhuman… distant’ and ‘very mechanic [whereas personalised medicine] is more personal’ (P6_CFG). Thus, participants generally appeared to be more comfortable and positive about ‘personalised medicine’:

Precision is harder to understand, and personalised medicine is easier to understand. Personally, precision sounds like a prestige concept and therefore personalised medicine will sound more ordinary. (P7_CFG).

1. Understandings of ‘personalised’ and ‘precision medicine’ after video

After watching the video, participants across the three groups identified ‘precision medicine’ as a more accurate or ‘comprehensive’ (P1_CFG) description of the research approach. One participant said precision medicine was a better descriptor because of the ‘use of DNA, individualised study and big data’ (P5_CFG). For participants, it sounded more scientific, professional and technologically sophisticated than ‘personalised medicine’:

Between the two, precision … sounds more relevant for a healthcare context … because when you talk of precision engineering is you’re engineering a medicine … when you talk about personalising, that is more like you personalise your phone cover, you personalise your product, you give the power of the choice to the consumers, which is not in this case. (P6_EFG).

While some participants changed their views following the video, saying that ‘precision medicine’ was more appropriate, a few of these participants still expressed a preference for the term ‘personalised medicine’ even though they conceded that ‘precision’ more aptly described the science.

Personally, I prefer personalised medicine but after watching the video, I think that precision medicine is more comprehensive in describing the treatment. (P1_CFG).

One participant explained, ‘I do not want to become a number’ (P6_CFG), and referred to the ‘mechanic’ and ‘scientific’ connotation of the term ‘precision medicine’. Another felt that ‘personalised medicine would be easier to understand’ (P3_CFG). One participant in the Malay group was dissatisfied with both terms:

… precision… that word is an awkward term … this is the first time we’ve heard of such a term. So if we announce it to the public, maybe they would be unsure of the meaning. ‘Personalised’ is also not that accurate [a descriptor] … (P4_MFG).

Throughout the discussions, participants returned to their unfamiliarity with the concept of PM. Though participants had watched the video, which described the basics of PM, they still had questions. Participants asked about the differences between current approaches to treatment and PM. They noted that patients have to be seen in person by the doctor, and the treatment subsequently prescribed is based on that consultation. ‘[W]hen we see the doctor, is it not precision treatment? I don’t really understand the difference.’ (P5_FG7). Moreover, current treatment is already targeted: ‘specifically target certain [illnesses] because we know the chemistry already inside the body… that’s how medicine is today.’ (P2_FG1). Some compared PM to current approaches to medicine, such as cancer chemotherapy.

There was some discussion about whether the ‘precision’ in PM refers to the medicine, the illness or the patient. For example, participants debated as to whether it would be ‘a type of treatment technique’ (P5_CFG), ‘targeting a specific gene’ (P2_EFG) or ‘specifically for one illness’ (P6_EFG). Some suggested alternative terms, such as ‘targeted medicine’ (P5_CFG), ‘customised’ or ‘menyesuaikan’ in Malay (P4_MFG) and ‘genetic medicine’ (P6_EFG) or ‘DNA medicine’ (P5_MFG) to make more explicit the reliance on genome data.

2. Views on sharing genomic data

Despite participants’ lack of familiarity with the term(s) or nature of PM, they were able to quickly grasp and engage with the ethical issues, tensions and trade-offs if genomic data had to be shared, for instance, with other doctors. Without prompting, participants were able to identify possible risks to security and privacy from a data breach and discriminatory harms arising from the misuse of data, particularly by insurance companies. Some participants noted that there had previously been data breaches at one of the three hospital clusters in Singapore (P10_EFG) and the national registry of HIV-positive patients (P5_MFG, P2_EFG). However, participants also suggested that assessment of these risks should be considered alongside the potential health and societal benefits of sharing data for worthwhile purposes, such as medical research. They referred to strategies to mitigate risks, including adequate oversight, ‘regulations’ (P4_EFG) and data security measures, such as de-identification. As a result of these risks, particularly the risks associated with uncertainty, some participants were more divided on the issue of sharing data: ‘There's too many unknown factors there’ (P2_EFG).

Participants also raised as an issue of concern the potential high costs and inequitable access to the benefits of PM. A number of them were concerned about the monetary cost, but cost also related to engagement, as this quotation makes clear:

Is it a price that rich people can afford to pay? Can ordinary citizens like us afford to as well? If I cannot afford it, whatever name you give it, will not matter to me. (P6_CFG).

For some, the idea of delivering PM sounded expensive and would only be available to the wealthy, but they suggested that it should ‘benefit everybody regardless of whether you are rich, poor, or regardless of race and all that’ (P4_EFG). Others were concerned about resource constraints and limitations of PM. They suggested that ‘if every patient [needs PM], then it is not feasible’ (P2_CFG), referring to the perceived burdens searching through databases would place on doctors’ time. Some suggested the value of doctors having access to genome sequencing data was limited unless they were trained or ‘knowledgeable’ in interpreting that information (P8_CFG).

## Discussion

Prior to watching the video, participants indicated preferences for the term ‘personalised medicine’ rather than ‘precision medicine’, as the former sounded more familiar. Their interpretations of the term ‘personalised medicine’ corresponded with prior research (Juengst et al. 2016) where this language is associated with conventional approaches to evidence-based medicine and patient-care. Similar to what we observed, Juengst et al. found that the terms ‘precision’ or ‘personalised’ medicine carry different connotations with the latter term carrying ‘the ideal of ‘individually tailored’ medicine’ (Juengst et al. 2016). In other research, scholars disagreed with the term ‘personalised medicine’, arguing that medicine has always been personalised and therefore the term does not adequately describe what is novel about this new form of genomic and data driven research (Ashley 2015; Lopez-Campos et al. 2014). Participants in our study shared this concern.

Many participants were unfamiliar with the term ‘precision medicine’ prior to watching the video. Even after watching the video, they asked for conceptual clarifications and some were trying to reach an understanding by comparing it to existing approaches to clinical treatment. Many of our participants were relatively highly educated, and we note that the term ‘precision’ holds certain meanings if no specific information is imparted to clarify the field to which it refers. For instance, ‘precision’ as defined in engineering and chemistry differs from the use of the term in precision medicine. Therefore, the possible opacity of the term ‘precision medicine’ has implications for the implementation of a PM programme, and highlights the need to engage in public communications to ensure clarity. Of note is the fact that participants gained a clearer understanding of the concept after they watched the video despite the term not featuring in the video. This could be evidence not only of areas of knowledge people already hold but also of the relative ease with which such complex concepts can be grasped without high levels of scientific knowledge.

Participants from the Malay group were unfamiliar with both terms ‘precision medicine’ and ‘personalised medicine’. The lack of familiarity with the term ‘precision medicine’ in the Malay group could, in part, also be due to difficulties encountered with translating the term into Malay. There was initial confusion with the term used to describe ‘precision’ (‘kepersisan’), and a participant reiterated the ‘awkwardness’ of the term after the video was shown. It is important to note that the term chosen was not considered inaccurate or poorly chosen, but that it was not commonly used. The Malay translator had issues with the term as well and there had been some discussion with other Malay-speaking colleagues before we decided on the translated term. This example demonstrates the complexities of cross language qualitative research as well as the challenges of clear and consistent public communication in multi-lingual contexts with different ethnic groups.

The educational video was intended to inform participants of key concepts and focus their discussion and our research did not set out to evaluate its use as an intervention. However, we note that the video was effective at communicating key ideas regarding precision medicine. Many participants changed their preferences after watching the video, saying that ‘precision medicine’ more accurately describes the concept and goals of PM. Though some participants thought ‘precision medicine’ was more appropriate after watching the video, a few still preferred the term ‘personalised medicine’. That a few participants retained their initial preference perhaps suggests the persistence of initial impressions or anchoring fallacy (Teovanović 2019; Tversky and Kahneman 1974), and therefore the importance of choosing terms with care. The reasons for the preferences were in tension—‘personalised medicine’ sounded familiar but ‘precision medicine’ seemed more accurate. Generally though, there was a sense that most of the participants did not feel strongly about one term or the other. Rather, our findings suggest that participants prefer terms that are more familiar but also accurately convey the concepts of PM.

Participants engaged in a sophisticated and nuanced discussion of the merits and risks of precision medicine research. Unprompted, participants raised most of the key ethical and governance issues addressed in the literature on precision medicine and data sharing, including: public benefit, commercialisation, impact on insurance, cost, equity, access, discrimination and personal health data security, all of which are addressed in a Nuffield Council on Bioethics (2015) report. Similar to a study by Budin-Ljøsne and Harris (2016), participants were concerned about the potential high cost of PM. Participants’ discussion on these points demonstrated a weighing of competing values and pragmatic acknowledgment that all research and data sharing entails some risk. Few existing empirical studies have captured the public’s efforts to evaluate these trade-offs (Ipsos-MORI 2016; Kim et al. 2017; Lysaght et al. 2020). Such a finding is of interest, as it indicates that members of the public already had a good understanding of the complex ethical concepts and deliberations involved in making decisions about the use of data in this area of medicine even if they were unfamiliar with PM specifically.

We do note that our research population was relatively highly educated (54.2% had a degree or post graduate qualification), young (most were under 37 years old), and some were familiar with and evidently interested in participating in research. Nationally, the proportion of Singaporean residents with an undergraduate degree or higher is 31.6% (Singapore Department of Statistics 2019). Effort was taken to ensure a wide spread of ages in our focus groups, but ensuring a nationally representative sampling was not our aim. Hence, further research with a more representative sample is necessary. It will be valuable to know whether a broader cross-section of the Singaporean population has a robust understanding of the ethical, legal, and social issues associated with data sharing in medicine, as this will inform public engagement strategies.

Scholars have noted the importance of inclusion and diversity in genomic research, ‘to the extent that any group abstains from participation, their members will be less able to share in the rewards precisely because their genetic and microbiomic samples are absent from the pool’ (Rhodes 2008, 38). There have been great efforts to increase participation and build genomic research capacity in Asia. Popejoy and Fullerton (2016) found that genome-wide association studies of participants with Asian ancestry rose from 3% in 2009 to 14% in 2016, and that 93% of these studies took place in Asian countries. Programmes such as China’s Precision Medicine Initiative (PMI) (Cyranoski 2016), The BioBank Japan Project (Nagai et al. 2017), and the planned Singapore’s National Precision Medicine (NPM) programme will boost the field of precision medicine in Asia. However, to fully realise the aims of these initiatives, broad public participation is crucial. The video had prompted observations by participants on the scarcity of Asian research data in genomics research and the value of improving Asian representation in PM.

Our study has demonstrated the impact of a short simplified explanatory video on fostering engagement and in-depth discussions about the ethical, legal and social implications of PM. Videos are an example of a communication aid that can effectively facilitate public dialogue around the ethical and social value of PM, by easing public unfamiliarity with PM, and by communicating the concept and goals of PM. However, as has amply been demonstrated (Suldovsky 2016), this educational component is simply a preliminary step in positively engaging with publics to ensure that public confidence in such programmes is forthcoming. Deeper, more meaningful public participation is essential to further establishing and maintaining the social licence for precision medicine, especially in the absence of specific consent (Minari et al. 2018; Xafis et al. 2019).

Some participants continued to have reservations about the sharing of genomic data. Our findings are therefore consistent with previous studies which highlight that public support of a complex and controversial scientific issue is often influenced by broader social factors, not simply a better understanding of the science (Bak 2001; Sturgis et al. 2010). In fact, participants in this research raised concerns about essential ethical issues such as equity of access, privacy considerations, and the potential threat of data breaches (Au Yong and Tham 2018; Chang et al. 2019); while also recognising the need to weigh the potential risks against the potential benefits arising from such programmes. This coheres with observations about important factors shaping public attitudes, which include the perceived social value of the research, the protection of privacy, harm minimisation and trust in institutions to store and use the data responsibly (Kalkman et al. 2019).

## Conclusion

Participants were unfamiliar with both terms—personalised or prevision medicine. In fact, participants of one ethnic group had not heard of either term. Participants were initially attracted to the term ‘personalised’ because it sounded more familiar and connoted standard healthcare interaction. After watching the educational video about PM the majority agreed that ‘precision medicine’ more accurately described genomic and data intensive medicine.

Some participants found both terms to be unsuitable to describe this field of medicine and research and offered new terms which they believed better represented the complex science involved. These findings suggest that the Singaporean public do not have a strong preference for either term and would be receptive to different terminology so long as the underlying goals and processes of PM are clearly described.

We did not aim to evaluate the video as an intervention. However, the value of readily accessible educational materials became evident because a single short video provided participants with a solid understanding of the science, as evidenced by subsequent nuanced discussions of the terms and the range of complex ethical and governance issues participants raised.

Participants demonstrated some prior knowledge of and sensitivity to pertinent ethical issues and an ability to consider and weigh opposing interests and values with respect to data sharing and genomic research. For example, they initiated debate about issues discussed extensively in the bioethics literature such as, for example, equity of access and privacy considerations. Additional research in the Singaporean context could clarify if this knowledge is more broadly shared across the population. If this were the case, there would be fertile ground for policy makers and researchers to engage in robust public discussions and exchanges about the merits and risks of a national PM programme.

## Data Availability

The data sets generated and/or analysed during the current study are not publicly available for reasons of personal privacy, but are available from the corresponding author on reasonable request.

## Footnotes

1 The video can be viewed at https://medicine.nus.edu.sg/cbme/research/trustworthy-governance-for-sharing-health-related-data/, under the ‘Videos’ tab. The video shown for this study was stitched together from Video A and the first part of Video B (until 1:42).

2 The Singapore-Cambridge General Certificate of Education A and O levels are the graduating examinations typically taken at 18 and 16 years. Diplomas are offered by polytechnics and arts institutions in Singapore as a more industry-oriented equivalent to the A levels. Advanced diplomas are designed to further specialisations for diploma or degree holders.
